# Nano-Clay Platelet Integration for Enhanced Bending Performance of Concrete Beams Resting on Elastic Foundation: An Analytical Investigation

**DOI:** 10.3390/ma16145040

**Published:** 2023-07-17

**Authors:** Mohammed Chatbi, Zouaoui R. Harrat, Mohammed A. Benatta, Baghdad Krour, Marijana Hadzima-Nyarko, Ercan Işık, Slawomir Czarnecki, Mohamed Bachir Bouiadjra

**Affiliations:** 1 Laboratoire des Structures et Matériaux Avancés dans le Génie Civil et Travaux Publics, Djillali Liabes University, Sidi Bel-Abbes 22000, Algeria; mohammed.chatbi@dl.univ-sba.dz (M.C.); zouaoui.harrat@dl.univ-sba.dz (Z.R.H.); bematif@gmail.com (M.A.B.); krour.bag@gmail.com (B.K.); mohamedbachirbouiadjra@gmail.com (M.B.B.); 2 Department of Civil Engineering, Josip Juraj Strossmayer University of Osijek, Vladimira Preloga 3, 31000 Osijek, Croatia; mhadzima@gfos.hr; 3 Faculty of Civil Engineering, Transilvania University of Brașov, Turnului Street No. 5, 500152 Brașov, Romania; 4 Department of Civil Engineering, Bitlis Eren University, 13100 Bitlis, Turkey; eisik@beu.edu.tr; 5 Department of Civil Engineering, Wroclaw University of Science and Technology, Wybrzeze Wyspianskiego 27, 50-370 Wroclaw, Poland

**Keywords:** nanotechnology, clay nano-platelets, nano-reinforced concrete, homogenization, quasi-3D beam theory, mechanical bending analysis, soil medium

## Abstract

Acknowledging the growing impact of nanotechnologies across various fields, this engaging research paper focuses on harnessing the potential of nano-sized materials as enhancers for concretes. The paper emphasizes the strategic integration of these entities to comprehensively improve the strength and performance of concrete matrixes. To achieve this, an analytical study is conducted to investigate the static behavior of concrete beams infused with different types of clay nano-platelets (NC’s), employing quasi-3D beam theory. The study leverages the effective Eshelby’s homogenization model to determine the equivalent elastic characteristics of the nanocomposite. The intricate interactions of the soil medium are captured through the use of a Winkler–Pasternak elastic foundation. By employing virtual work principles, the study derives equations of motion and proposes analytical solutions based on Navier’s theory to unravel the equilibrium equations of simply supported concrete beams. The results shed light on influential factors, such as the clay nano-platelet type, volume percentage, geometric parameters, and soil medium, providing insights into the static behavior of the beams. Moreover, this research presents previously unreported referential results, highlighting the potential of clay nano-platelets as reinforcements for enhancing structural mechanical resistance.

## 1. Introduction

Concrete, a widely used building material known for its cost-effectiveness, high strength, and durability [1], is susceptibility to brittleness, cracking, and failure under mechanical stresses [2]. To address these concerns, various reinforcement techniques have been employed, including the incorporation of steel bars [3], fibers [4], and more recently, nanoparticles [5]. Notably, the use of nanoparticles has demonstrated significant potential in enhancing the mechanical properties of concrete, such as its compressive and tensile strength, fracture toughness, and ductility [6].

Nanoparticles, characterized by their size ranging from 1 to 100 nanometers, possess more unique physical, chemical, and mechanical properties compared to those of their bulk counterparts [7]. When they are incorporated into concrete, nanoparticles facilitate improved interfacial bonding between the particles and the matrix, resulting in an enhanced load transfer and increased resistance to crack propagation [8]. Furthermore, nanoparticles offer additional benefits, such as serving as a protective barrier against the ingress of harmful ions, thus mitigating environmental factors like corrosion [9]. The incorporation of nanoparticles into concrete represents a versatile range of methods used to achieve optimal dispersion and integration, each tailored to specific considerations [10]. These methods include direct mixing, surface functionalization, and electrostatic attraction, each offering unique advantages and considerations [11].

Direct mixing entails the addition of nanoparticles directly into the concrete mixture during the mixing process. This approach requires meticulous control of the mixing conditions to ensure the uniform distribution of nanoparticles throughout the concrete matrix, ultimately influencing the overall properties of the composite material [11]. Alternatively, surface functionalization involves modifying the surface chemistry of nanoparticles to enhance their compatibility with the concrete matrix. By optimizing the interfacial bonding between nanoparticles and the surrounding matrix, surface functionalization can significantly improve the mechanical properties and performance of the resulting composite material [12]. Another intriguing approach is the use of electrostatic attraction, which involves harnessing the power of electric fields to guide and disperse the nanoparticles onto the surface of cement particles. This method facilitates the controlled dispersion of nanoparticles within the concrete matrix, enhancing their uniform distribution and overall effectiveness [13].

In recent years, nanoparticles have emerged as a popular choice for reinforcing civil engineering structures, with a particular focus on the remarkable impact of silica dioxide (SiO_2_) nanoparticles on the mechanical behavior of impregnated concrete. Extensive research has been conducted to unravel their potential, shedding light on various aspects of their influence. For instance, Shokravi [14] delved into the vibration analysis of silica nanoparticle-reinforced concrete beams, taking into account the intriguing effects of agglomeration. Zamanian Mohammad and Bidgoli [15] explored the impact of agglomeration on the buckling behavior of embedded concrete columns reinforced with SiO_2_ nanoparticles. Heidari and Tavakoli [16] carried out a numerical study, examining the vibration response of concrete beams reinforced with SiO_2_ nanoparticles. In a similar vein, Nasihatgozar [17] conducted a meticulous investigation, studying the buckling analysis of concrete beams containing SiO_2_ nanoparticles using numerical approaches. Meanwhile, Jassas et al. [18] conducted the forced vibration analysis of concrete slabs reinforced with agglomerated SiO_2_ nanoparticles employing advanced numerical methods. More recently, Harrat et al. [19] presented a comprehensive study on the static behavior of nano-SiO_2_-based concrete beams resting on an elastic foundation. Similarly, Chatbi et al. [20] investigated the bending analysis of nano-SiO_2_-reinforced concrete slabs supported by an elastic foundation. These studies collectively established that the incorporation of SiO_2_ nanoparticles in concrete yields increased their mechanical resistance, reduced the deflections, diminished the stresses, and lowered the vibrational natural frequencies.

Various types of nanoparticles have been explored for their potential use in concrete, showcasing the versatility and wide-ranging benefits of these innovative materials. Zinc Oxide nanoparticles, for example, have been investigated by Arbabi et al. [21] for reinforcing concrete columns subjected to electric fields via the careful analysis of their buckling behavior. In a different study, Ghahari et al. [22] studied the effect of ZnO nanoparticles on the thermoelectric properties of cement composites for waste heat harvesting. Moreover, TiO_2_ nanoparticles have garnered attention due to their impact on the compressive strength of concrete [23]. Tabatabaei [24] conducted a thorough investigation and reached the conclusion that TiO_2_ nanoparticles exhibit promising capabilities for reducing the environmental pollution of concrete structures. On the other hand, Seifan et al. [25] demonstrated that the utilization of iron oxide nanoparticles (Fe_2_O_3_) has been instrumental in the advancement of bio-reinforced self-healing concrete. Their findings showcase the immense potential of these nanoparticles in enhancing the mechanical properties and self-healing capabilities of this material. Recently, Harrat et al. [26] conducted an analytical study on the thermoelastic bending of iron oxide-impregnated concrete slabs. Their research revealed that the utilization of this type of reinforcement can significantly enhance the mechanical flexural performance by up to 45 percent. However, it was observed that the flexural performance against thermomechanical loads decreased by approximately 10 percent. The influence of Al_2_O_3_ nanoparticles on the compressive strength and workability of blended concrete has also been investigated by Nazari et al. [27]. Similarly, the mechanical and microstructural characterization of Al_2_O_3_ nanoparticle-modified cement concrete has been examined by Meddah et al. [28], shedding light on their potential in improving the material’s performance.

The utilization of clay nanoparticles as a concrete reinforcement remains an area that has not been extensively explored, with limited research having been conducted in this captivating field. Hosseini et al. [29] investigated the effects of nano-clay particles on the short-term properties of self-compacting concrete, providing valuable insights into their impact. Furthermore, Niaki et al. [30] explored the mechanical and thermal properties of basalt fiber- and nano-clay-reinforced polymer concrete, revealing the potential of these combinations. Furthermore, the implications of nanoparticle use in construction materials and other applications have been discussed by Mohajerani et al. [31], highlighting the broad applicability of this technology. To comprehensively assess the potential of clay and titanium dioxide nanoparticles in mortar and concrete, state-of-the-art analysis was performed by Bunea et al. [32], providing valuable insights into their usage. Collectively, these studies demonstrate the positive impact of clay nanoparticles as a concrete reinforcement, suggesting the development of a new material with enhanced strength, elasticity, and durability properties. The exploration of such innovative reinforcements holds great promise for advancing the field of concrete engineering.

The analytical modeling of load-bearing elements, such as beams and plates, is highly dependent on the mathematical tools used, specifically deformation theories. A recent notable advancement in this area is the quasi-3D deformation theory [33,34]. Unlike traditional theories that primarily consider bending and shear components in the transverse displacement of element sections [35], the quasi-3D deformation theory incorporates the additional effect of stretching that extends throughout the thickness of the elements. This becomes particularly significant when one is analyzing thick structures [36,37]. The application of deformation theories to the prediction of the mechanical behavior of composite elements is highly valuable. For instance, Babaei et al. [38] conducted a study on the vibrational behavior of thermally pre-/post-buckled functionally graded carbon nanotube-reinforced concrete beams. This study involved the use of three different beam theories: first-order, third-order, and sinusoidal beam theories. Similarly, Kiani and Krzysztof [39] explored the free vibrations of graphene platelet-reinforced composite skew plates that were resting on a point support. The quasi-3D beam theory has been recently employed in numerous investigations to examine the mechanical behaviors and responses of composite plates and beams. For example, Xin and Kiani [40] conducted a study on the vibration characteristics of arbitrary, thick sandwich beams with a metal foam core resting on an elastic medium. They considered the thickness stretching and accounted for non-uniform through-the-thickness shear strain. Jafari and Kiani [41] utilized a four-variable shear and normal deformable quasi-3D beam model to analyze the free and forced vibrations of composite beams reinforced with graphene under a moving load. Wang and Kiani [42] explored the effects of initial compression/tension, foundation damping, and a Pasternak medium on the dynamics of shear and normal deformable beams reinforced with graphene platelets (GPLs) under a moving load. Afshari et al. [43] conducted vibration analyses and investigated the size-dependent buckling of GNP-reinforced microplates based on the quasi-3D sinusoidal shear deformation theory. Additionally, Adim et al. [44] studied the effects of thickness stretching in FGM plates using a quasi-3D higher-order shear deformation theory. These studies collectively highlight the reliability and applicability of the quasi-3D deformation theory in various structural analyses.

In the field of concrete reinforcement, clay nanoparticles have emerged as a promising resource. However, it is surprising that the scientific community has not extensively explored their influence on concrete bending behavior using rigorous analytical modeling. In recognition of this critical knowledge gap, through our research, we embark on a comprehensive exploration aimed at unraveling the intricacies associated with the effective utilization of clay nanoparticles as reinforcements in concrete beams.

We endeavor to shed light on the intricacies of the analytical approach via meticulous analysis. By delving into the profound impact of key parameters, such as the volume proportion of diverse clay nano-reinforcements within the concrete matrix, the geometric ratios of the beam, the constants of the soil medium, and the load patterns, we uncover their intricate roles in shaping the static behavior of the beam and influencing the overall structural performance. To ensure the utmost reliability of our findings, we employ quasi-3D beam deformation theory, enabling us to accurately simulate the behavior of the reinforced concrete beams.

## 2. Brief Overview on Nano-Clay Cementitious Materials Composites

Nano-sized clay platelets, which are composed mostly of layered mineral silicates, can be classified into various categories based on their chemical compositions and morphologies, such as bentonite, montmorillonite, kaolinite, halloysite, and hectorite. Harraz et al. [45] outlined these classification types and provided a schematic diagram illustrating their structures.

In Table 1, the composition of nano-clays reveals that the highest proportion is attributed to silicate dioxide (SiO_2_), followed by aluminum oxide (Al_2_O_3_). Both of these components play a crucial role in enhancing the hydration rate and setting time of cement. Silicate dioxide is a key ingredient in cementitious materials and contributes to their strength and durability. Aluminum oxide, on the other hand, can act as a pozzolan, reacting with calcium hydroxide during hydration to form additional cementitious compounds. The presence of higher amounts of SiO_2_ and Al_2_O_3_ in nano-clays suggests their potential for positively influencing the cement properties [46].

However, nano-clays have a low content of calcium oxide (CaO), as indicated in Table 1. CaO is a major constituent in ordinary Portland cement and contributes to the development of early strength in concrete. Nevertheless, the high proportion of SiO_2_ and Al_2_O_3_ in nano-clays can still have a beneficial impact on cement hydration and the setting time, potentially compensating for the lower CaO content generated via the use of other mechanisms.

In Table 2, the physical properties of clays and nano-clays are presented. It is evident that there is a significant increase in the surface area when the clay size transitions from the micron scale to the nanometer scale. The data show that the surface area of the clay increases from a factor of 2 to 8 when the particle size decreases from microns to nanometers.

The substantial increase in surface area has significant implications for the properties and behavior of nanoscale clays. The larger surface area provides more sites for adsorption, enhanced reactivity, and increased interfacial interactions with surrounding substances. These properties can have a pronounced influence on various applications, such as catalysis, adsorption processes, the reinforcement of composite materials, and surface modifications [49].

### 2.1. Physical Characterisation of Nano-clay–Cementitious Materials Composites

**Figure 1:**
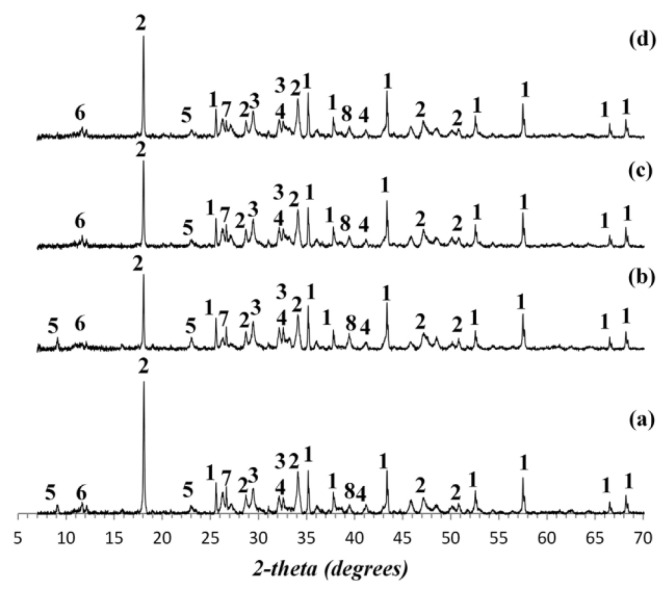
XRD patterns of: (**a**) cement paste, cement nanocomposite containing: (**b**) 1 wt% calcined NC, (**c**) 2 wt% calcined NC, and (**d**) 3 wt% calcined NC [53]. Numbers indicate: 1: Corundum [Al_2_O_3_] phase; 2: Portlandite [Ca(OH)_2_] phase; 3: Tri-calcium silicate [C_3_S] phase; 4: Di-calcium silicate [C_2_S] phase; 5: Ettringite phase; 6: Gypsum phase; 7: Quartz phase; 8: Calcite phase.

Regarding the physical characterization of nano-clay–cementitious materials composites, Hakamy et al. [53] utilized Quantitative X-ray Diffraction Analysis (QXDA) to investigate the properties of clay–cement nanocomposites. The study focused on analyzing the composition and phase content of cement paste and cement nanocomposite samples containing nano-clay. The experimental setup involved using Ordinary Portland cement (OPC) samples partially substituted with 1%, 2%, and 3% of NC by weight of OPC, as illustrated in Figure 1.

The X-ray diffraction measurements were conducted with a D8 Advance Diffractometer (Bruker-AXS) using Cu Ka (λ = 1.5406 Å) radiation. The scanning range for the diffractometer was from 7° to 70° (2θ) with a scanning rate of 0.5°/min. Quantitative X-ray Diffraction Analysis (QXDA) with Rietveld refinement was conducted with Bruker DIFFRAC^plus^ TOPAS software associated with the International Centre for Diffraction Data PDF-4 2013 database. Corundum [Al_2_O_3_] was chosen to serve as an internal standard. The samples for QXDA were prepared by mixing a dry weight of 3.0 g of cement paste or cement nanocomposite paste, with 0.33 g of Corundum [Al_2_O_3_] as the internal standard [54].

As evident from the data presented in Table 3, the incorporation of 1 wt% nano-clay (NC) into the cement nanocomposite resulted in a reduction of the amount of portlandite, decreasing it to 13.8 wt% compared to that of approximately 16.8% in the cement paste. In addition, the quantities of C3S (1.3 wt%) and C2S (4.4 wt%) in the ordinary cement were elevated to 1.5 wt% for C3S and 6.1 wt% for C2S upon the inclusion of 1 wt% NC in the cement mixture. The decrease in portlandite content may be a consequence of the pozzolanic reaction between the nano-clay and calcium hydroxide (portlandite) present in the cement. Overall, the observed changes in the composition of the cement nanocomposite, including the decrease in portlandite content and the increase in C3S and C2S levels, can be attributed to the pozzolanic reaction and the interaction between nano-clay and the cementitious phases.

### 2.2. Mechanical Testing Methods for Clay–Cementitious Composite Materials

The evaluation of the mechanical performance of clay–concrete composites involved various testing methods, including compressive strength testing and flexural strength testing. Several studies have reported that the incorporation of nano-clay (NC) in cementitious materials results in an improved compressive and tensile strength [55,56]. The effect of incorporating NC on the mechanical strengths of ordinary concrete at 28 days is summarized in Table 4.

Table 4 clearly demonstrates the considerable impact of incorporating nano-clay (NC) in ordinary concrete mixtures, particularly in terms of compressive strengths. It reveals a substantial increase in compressive strength with the addition of a specific percentage of NC [48]. For the sample of concrete in which the water-to-binder ratio (w/b) is 0.53 and for a concentration of 10% NC, Alsallami et al. [58] found a significant amelioration in the compressive strength of up to 63%.

Furthermore, Table 5 presents compelling results indicating that the inclusion of nano-clay (NC) in concretes leads to a significant improvement in the splitting tensile strength. It is noteworthy from Table 5 that when using 10% of nano-clay reinforcements in concrete with a water-to-binder ratio of 0.53, there is a substantial increase in tensile strength, reaching up to 46.6%, as reported by Ibrahem et al. [59].

### 2.3. Durability Testing Methods for Clay–Cementitious Composite Materials

The durability of clay–concrete composites was evaluated using a range of testing methods, including water absorption and chloride ion penetration tests. These tests aimed to assess the ability of the composites to resist the ingress of water and the penetration of chloride ions. Water absorption testing helps determine the porosity and permeability of the composites, which are important factors affecting their durability. On the other hand, chloride ion penetration testing is performed to measure the resistance of the composites to chloride ion ingress, as chloride ions can cause structural deterioration. By evaluating these durability parameters, the performance and long-term serviceability of clay–concrete composites can be assessed and optimized.

Table 6 provides a summary of the effects of nano-clay (NC) on the coefficient of capillary water absorption in cementitious materials. In general, these include an increase in the content of NC results in a decrease in the water absorption coefficient. These indicate that the incorporation of nano-clay in the cementitious matrix helps to reduce the permeability of the material and enhance its resistance to water absorption [60]. This can be attributed to the improved pore structure resulting from the pozzolanic reaction of the nano-clay, which leads to the production of additional C-S-H gel. This gel fills the micro-pores between the cement particles, reducing the pathways for water penetration and absorption in the cement paste [61]. The geometry and size of the pore system also play a crucial role in the mechanism of capillary water absorption [62].

When it comes to the sulfate ion attack on concrete and cementitious materials reinforced with clay nanoparticles, there is currently a limited amount of research specifically addressing this particular case. However, in recent years, a growing body of research has shown that the incorporation of alternative nanomaterials, such as silica nanoparticles, can greatly improve the sulfate resistance of cement-based materials. This improvement is attributed to the reduction of microstructural pores. These studies have shown that the inclusion of silica nanoparticles can lead to improved sulfate resistance, particularly during the early stages of concrete curing [65,66].

Our research article is driven by the compelling experimental data that highlight the remarkable properties of nano-clays and their ability to enhance the overall behavior of concretes. Building upon these findings, our study aims to comprehensively explore the potential of montmorillonite, kaolinite, illite, and hectorite nano-clays, unveiling novel referential findings that have not been reported before.

The primary focus of our research is to analytically model the bending behavior of concrete beams that are reinforced with varying types and proportions of clay nanoparticles. To achieve this, we employ analytical homogenization techniques, which enable us to unravel the intricate interplay between the presence of NCs and the structural performance of the beams.

## 3. Homogenization Model

The integration of nanoparticles with diverse properties into a concrete matrix introduces a fascinating dimension of microstructural heterogeneity. The resulting effective elastic properties of this concrete-reinforcement combination are of great interest, and in this pursuit, the renowned Eshelby’s homogenization model [67], which was developed in 1957, is a powerful tool.

**Figure 2:**
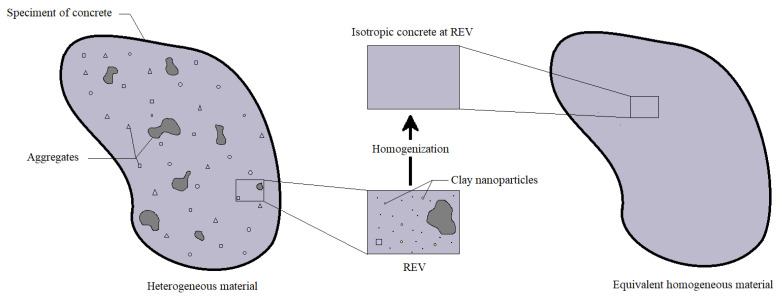
Homogeneity process of a concrete matrix incorporated with nano-sized clay inclusions.

Within the framework of this stochastic model, the material is conceptualized as a collective assembly of small particles or inclusions, sharing uniform size and shape characteristics. Specifically, in our case, the particles take the form of disc-shaped clay nanoparticles, gracefully occupying a designated sub-volume referred to as *V_r_*. These carefully chosen nanoparticles are randomly dispersed throughout a continuous matrix. This matrix, in its entirety, occupies the complementary volume known as *V_m_*, which is assumed to possess infinite extent and homogeneity, representing the concrete medium (see Figure 2).

By embracing Eshelby’s homogenization model, we can gain deeper insights into the behavior of this composite material. It allows us to calculate the effective elastic properties by replacing the intricate microstructural assembly with an equivalent homogeneous material that exhibits the same overall response. To achieve this, the stresses and strains within a representative volume element (RVE) are meticulously analyzed, utilizing the elastic properties of both the matrix and the included nanoparticles, while considering the geometric attributes of the RVE itself, as Clyne notes [68].

In terms of predicting the properties of a matrix that is reinforced with nano-sized inclusions. Eshelby’s homogenization model has demonstrated accurate predictions for inclusions in the shape of oblate and prolate spheroids when their volume fractions are below 30%, as Hull notes [69]. In our study, we consider the nanoparticles to have a disc-shaped form. The stiffness tensor *C^T^* for the nanocomposite is then derived using the following Equation (1):

(1) $$C^{T}={\left(C_{m}^{-1}-V_{r}{\left\{\left(C_{r}-C_{m}\right)\left[S-V_{r}\left(S-I\right)\right]+C_{m}\right\}}^{-1}\left(C_{r}-C_{m}\right)C_{m}^{-1}\right)}^{-1}$$

where *I* is the identity matrix, and *C_m_* and *C_r_* are the stiffness tensors for the concrete matrix and the nano-reinforcement, respectively. As mentioned earlier, the volume fraction of the matrix and reinforcement are denoted by *V_m_* and *V_r_*, respectively. Additionally, Eshelby’s tensor *S* is related to the Poisson ratios of the nanoparticles.

For both isotropic materials, the stiffness’s *C_m_* and *C_r_* are expressed in Equation (2) as:

(2a) $$C_{11}=\frac{\left(1-\upsilon \right)E}{\left(1+\upsilon \right)\left(1-2\upsilon \right)}$$

(2b) $$C_{12}=\frac{\upsilon E}{\left(1+\upsilon \right)\left(1-2\upsilon \right)}$$

(2c) $$C_{22}=\frac{\left(1-\upsilon \right)E}{\left(1+\upsilon \right)\left(1-2\upsilon \right)}$$

(2d) $$C_{44}=C_{55}=C_{66}=\frac{E}{\left(1+\upsilon \right)}$$

In which *E* is the young’s modulus of either the concrete matrix or the nanoparticles reinforcement, and *υ* denotes the Poisson’s ratio. Indexes 1, 2, and 3 conform to the *x*, *y*, and *z* directions of the composite Cartesian co-ordinate system, respectively.

For reinforcement with platelets, Eshelby’s tensor *S* is given in Equation (3) as: (Binns [70])

(3) $$S=\left[\begin{array}{cccccc} S_{1111} & S_{1122} & S_{1133} & S_{1123} & S_{1113} & S_{1112}\\ S_{2211} & S_{2222} & S_{2233} & S_{2223} & S_{2213} & S_{2212}\\ S_{3311} & S_{3322} & S_{3333} & S_{3323} & S_{3313} & S_{3312}\\ S_{2311} & S_{2322} & S_{2333} & S_{2323} & S_{2313} & S_{2312}\\ S_{1311} & S_{1322} & S_{1333} & S_{1323} & S_{1313} & S_{1312}\\ S_{1211} & S_{1222} & S_{1233} & S_{1123} & S_{1213} & S_{1212} \end{array}\right]$$

where the components of Eshelby’s tensor *S* are described in Equations (4a) and (4b) as:

(4a) $$\begin{array}{l} S_{1111}=S_{2222}=0\;;\;S_{3333}=1\\ S_{1122}=S_{1133}=S_{2233}=S_{2211}=0\;;\;S_{3311}=S_{3322}=\frac{\upsilon_{r}}{\left(1-\upsilon_{r}\right)}\\ S_{1212}=0\;;\;S_{1313}=S_{2323}=1/2 \end{array}$$

For all the others components:

(4b) $$S_{ijkl}=0$$

Here, *υ_r_* denotes the Poisson’s ratio of nano-platelets reinforcements.

## 4. Mathematical Modelling of Beams

**Figure 3:**
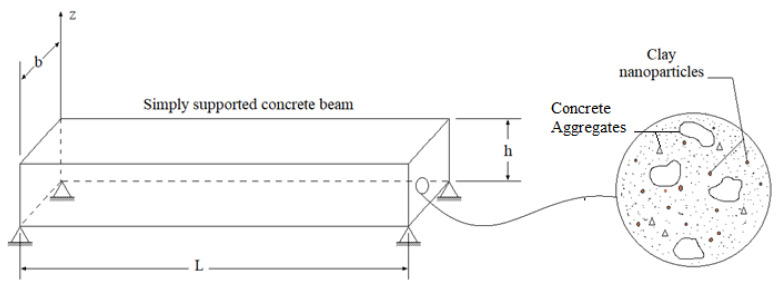
Geometry of a simply supported concrete beam reinforced with clay nano-platelets.

In the following Section, the analytical formulation of the nano-clay-reinforced concrete beam based on the kinematic and physical assumptions of the refined quasi-3D theory of Thuc Vo et al. [71] is presented. A simply supported reinforced concrete beam is considered to have a length ‘*L*’, width ‘*b*’, and total thickness ‘*h*’, as illustrated in Figure 3.

The impregnated clay nano-platelets are assumed to be randomly arranged in the concrete matrix, as shown in Figure 3. The coordinate system noted (*x*, *y*, and *z*), on which *z* is placed, is in the median plane of the beam.

0≤x<L ; 0≤y<b ; −h/2≤z<h/2

### 4.1. Kenimatics

In order to include the thickness stretching effect in the bending analysis of our nanoparticle-reinforced concrete beam, the total transverse displacement *u_3_* is assumed to be divided into 3 components: bending (*w_b_*), shear (*w_s_*), and the thickness stretching effect (*w_z_*) (Huu-Tai Thai et al. [72]). Thus, the transverse displacement *u_3_* and the in-plane axial displacement *U* of a material point located at (*x*, *y,* and *z*) in the beam are described in Equation (5) according to the refined quasi-3D theory as follows:

(5a) $$u_{1}(x,z)=u_{0}(x,z)-z\frac{\partial w_{b}(x,z)}{\partial x}-f(z)\frac{\partial w_{s}(x,z)}{\partial x}$$

(5b) $$u_{3}(x,z)=w_{b}\left(x,t\right)+w_{s}\left(x,t\right)+g(z)w_{z}(x,t)$$

At this point, *u_0_* and *v_0_* signify the displacement functions of the mid-planes of the beam. *f(z)* expressed in Equation (6) is the odd shape function used to expresses the distribution of shear strains throughout the beam’s depth:

(6) $$f\left(z\right)=z\left(\frac{1}{4}-\frac{5z^{2}}{3h^{2}}\right);\;g\left(z\right)=1-\frac{\partial f(z)}{\partial z}$$

The linear strains components associated with the quasi-3D displacement equations are given in Equation (7):

(7a) $$\varepsilon_{x}={\frac{\partial u_{1}}{\partial x}=\varepsilon }_{x}^{0}+zk_{x}^{b}+f\left(z\right)k_{x}^{s}$$

(7b) $$\varepsilon_{z}=\frac{\partial u_{3}}{\partial z}=\frac{\partial g\left(z\right)}{\partial z}w_{z}(x)$$

(7c) $$\gamma_{xz}=\frac{\partial u_{3}}{\partial x}+\frac{\partial u_{1}}{\partial z}=\left(1-\frac{\partial f(z)}{\partial z}\right)\gamma_{xz}^{s}=g(z)\gamma_{xz}^{s}.$$

where:

(8) $$\varepsilon_{x}^{0}=\frac{\partial u_{0}}{\partial x};\;k_{x}^{b}=-\frac{{\partial^{2}w}_{b}}{\partial x^{2}};\;k_{x}^{s}=-\frac{\partial^{2}w_{s}}{\partial x^{2}};\;\gamma_{xz}^{s}=\frac{\partial w_{s}}{\partial x};\;g(z)=1-\frac{\partial f(z)}{\partial z}.$$

For normal and shear stresses of the nanoparticle-reinforced concrete beam, the constitutive equations can be expressed as Equation (9):

(9) $$\left\{\begin{array}{c} \sigma_{x}\\ \sigma_{z}\\ \tau_{xz} \end{array}\right\}=\left[\begin{array}{ccc} C_{11}^{T} & C_{13}^{T} & 0\\ C_{13}^{T} & C_{33}^{T} & 0\\ 0 & 0 & C_{11}^{T} \end{array}\right]\left\{\begin{array}{c} \varepsilon_{x}\\ \varepsilon_{z}\\ \gamma_{xz} \end{array}\right\}$$

Herein, $C_{ij}^{T}$ are the reduced elastic constants of the concrete-reinforcement equivalent system, which can be obtained using Eshelby’s homogenization model.

### 4.2. Equations of Motion

In this analysis, the principle of the virtual work is applied in order to provide the equations of motion of the nano-reinforced beam:

(10) $$\int_{t1}^{t2}\left({\delta U}_{b}+{\delta W}_{k}+\delta \Phi \right)\partial t=0$$

where, *δU_b_* and *δW_k_* are the virtual variation in the internal strain energy of the beam and the elastic foundation, respectively, while *δΦ* is the virtual work performed by external bending forces.

Firstly, the expression of the virtual strain energy performed by the beam can be depicted as follows in Equation (11):

(11) $${\delta U}_{b}=\int_{0}^{L}\int_{-h/2}^{h/2}\left(\sigma_{x}\delta \varepsilon_{x}+\sigma_{z}\delta \varepsilon_{z}+\tau_{xz}\delta \gamma_{xz}\right)dAdz$$

Submitting Equation (7) into Equation (11) gives Equation (12) for the internal strain energy:

(12) $${\delta U}_{b}=\int_{0}^{L}\left(N\frac{\partial \delta u_{0}}{\partial x}-M_{b}\frac{\partial^{2}\delta w_{b}}{\partial^{2}x}-M_{s}\frac{\partial^{2}\delta w_{s}}{\partial^{2}x}+Q\frac{\partial \delta w_{s}}{\partial x}-R_{z}\delta w_{z}\right)dx$$

In Equation (12), the stress resultants (*N, M_b_*, *M_s_*, *Q*, and *R_z_*) represent the following quantities:

- ‘*N*’ represents the normal force in a structural element;
- ‘*M_b_*’ denotes the bending moment about the beam’s local y-axis;
- ‘*M_s_*’ indicates the shear force acting along the beam’s local z-axis;
- ‘*Q*’ represents the torsional moment applied to the beam;
- ‘*R_z_*’ represents the rotational moment about the member’s local z-axis.

where:

(13a) $$N=\int_{-h/2}^{h/2}\sigma_{x}bdz$$

(13b) $$M_{b}=\int_{-h/2}^{h/2}{z\sigma }_{x}bdz$$

(13c) $$M_{s}=\int_{-h/2}^{h/2}f(z)\sigma_{x}bdz$$

(13d) $$Q=\int_{-h/2}^{h/2}g(z)\tau_{xz}bdz$$

(13e) $$R_{z}=\int_{-h/2}^{h/2}\frac{\partial g(z)}{\partial z}\sigma_{z}bdz$$

By substituting Equation (8) into Equation (9) and the results into Equation (13), one obtains the stress resultants in form of material stiffness and displacement components, and the expressions are given as Equation (14):

(14a) $$N=A\frac{\partial u_{0}}{\partial x}-B\frac{\partial^{2}w_{b}}{\partial x^{2}}-B_{s}\frac{\partial^{2}w_{s}}{\partial x^{2}}+L_{z}w_{z}$$

(14b) $$M_{b}=B\frac{\partial u_{0}}{\partial x}-D\frac{\partial ²w_{b}}{\partial x²}-D_{s}\frac{\partial ²w_{s}}{\partial x²}+Y_{z}^{b}w_{z}$$

(14c) $$M_{s}=B_{s}\frac{\partial u_{0}}{\partial x}-D_{s}\frac{\partial^{2w_{b}}}{\partial x^{2}}-H_{s}\frac{\partial^{2w_{s}}}{\partial x^{2}}+Y_{z}^{s}w_{z}$$

(14d) $$Q=A_{s}\left(\frac{\partial w_{s}}{\partial x}+\frac{\partial w_{z}}{\partial x}\right)$$

(14e) $$R_{z}=L_{z}\frac{\partial u_{0}}{\partial x}-Y_{z}^{b}\frac{\partial^{2w_{b}}}{\partial x^{2}}-Y_{z}^{s}\frac{\partial^{2w_{s}}}{\partial x^{2}}+Z_{33}w_{z}$$

In which (*A*, *B*, *D*, *As*, *Bs*, *Ds*, and *Hs*) and (*Lz*, *Y^b^_z_*, *Y^s^_z_*, and *Z_33_*) are the beam stiffness, which are defined in Equation (15):

(15a) $$\left\{A,B,D,B_{s},D_{s},H_{s}\right\}=\int_{-h/2}^{h/2}\left\{1,z,z^{2},f\left(z\right),zf\left(z\right),f(z)²\right\}C_{ij}^{T}bdz$$

(15b) $$\left\{A_{s}\right\}=\int_{-h/2}^{h/2}\left\{g(z)²\right\}C_{ij}^{T}bdz$$

(15c) $$\left\{L_{z},Y_{z}^{b},Y_{z}^{s},Z_{33}\right\}=\int_{-h/2}^{h/2}\frac{\partial g(z)}{\partial z}\left\{1,z,f(z),\frac{\partial g(z)}{\partial z}\right\}C_{ij}^{T}bdz$$

In addition, the load–displacement formula between the concrete beam and the supporting foundation is expressed in Equation (16) by introducing the parameters (*K_w_* and *K_s_*) of the Winkler/Pasternak foundation model:

(16) $$\delta W_{k}=-\int_{L}^{}\int_{-h/2}^{h/2}\left(K_{w}\left(w_{b}+w_{s}+g(z)w_{z}\right)-K_{s}\left(\frac{\partial ²w_{b}}{\partial x²}+\frac{{\partial ²w}_{s}}{\partial x²}+g(z)\left(\frac{{\partial ²w}_{z}}{\partial x²}\right)\right)\right)dxdz$$

Herein, $\delta W_{k}$ is the strain caused by the elastic foundation reaction per unit area, and *K_s_* and *K_w_* are the transverse and shear and spring stiffnesses of the Winkler/Pasternak foundation, respectively, whereas without the elastic foundation, *K_w_ = K_s_ = 0*.

As for the nano-reinforced concrete beam subjected to mechanical bending loads ‘‘*q*’’, the external virtual work under those loads is described in Equation (17):

(17) $$\delta \Phi =-\int_{L}^{}\int_{-h/2}^{h/2}q\left(\delta w_{b}+\delta w_{s}+g(z)\delta w_{z}\right)dxdz$$

By combining Equations (12), (16), and (17) into Equation (10), and then integrating the coefficients of *δu_0_*, *δw_b_*, *δw_s_*, and *δw_z_*, the following equations of motion associated with the quasi-3D beam theory are obtained (Equation (18)):

(18a) $$\delta u_{0}\;:\;\frac{\partial N}{\partial x}=0$$

(18b) $$\delta w_{b}\;:\;\frac{\partial^{2}M_{b}^{}}{\partial x^{2}}+q-\left(K_{w}\left(w_{b}+w_{z}+{gw}_{z}\right)-K_{s}\frac{\partial^{2}\left(w_{b}+w_{z}+{gw}_{z}\right)}{\partial x^{2}}\right)=0$$

(18c) $$\delta w_{s}\;:\;\frac{\partial^{2}M_{s}^{}}{\partial x^{2}}+\frac{\partial Q}{\partial x}+q-\left(K_{w}\left(w_{b}+w_{z}+{gw}_{z}\right)-K_{s}\frac{\partial^{2}\left(w_{b}+w_{z}+{gw}_{z}\right)}{\partial x^{2}}\right)=0$$

(18d) $$\delta w_{z}\;:\;\frac{\partial Q}{\partial x}-R_{z}+g\left(z\right)q=0$$

where:

(19a) $$A\frac{\partial ²u_{0}}{\partial x²}-B\frac{\partial^{3}w_{b}}{\partial x^{3}}-B_{s}\frac{\partial^{3}w_{s}}{\partial x^{3}}+L_{z}\frac{\partial w_{z}}{\partial z}=0$$

(19b) $$B\frac{\partial^{3}u_{0}}{\partial x^{3}}-D\frac{\partial^{4}w_{b}}{\partial x^{4}}-D_{s}\frac{\partial^{4}w_{s}}{\partial x^{4}}+Y_{z}^{b}\frac{\partial^{2}w_{z}}{\partial x^{2}}+Q-\left(K_{w}\left(w_{b}+w_{z}+{gw}_{z}\right)-K_{s}\frac{\partial^{2}\left(w_{b}+w_{z}+{gw}_{z}\right)}{\partial x^{2}}\right)=0$$

(19c) $$B_{s}\frac{\partial^{3}u_{0}}{\partial x^{3}}-D_{s}\frac{\partial^{4}w_{b}}{\partial x^{4}}-H_{s}\frac{\partial^{4}w_{s}}{\partial x^{4}}+A_{s}\left(\frac{\partial^{2}w_{s}}{\partial x^{2}}+\frac{\partial^{2}w_{z}}{\partial x^{2}}\right)+Y_{z}^{s}\frac{\partial^{2}w_{z}}{\partial x^{2}}+Q-\left(K_{w}\left(w_{b}+w_{z}+{gw}_{z}\right)-K_{s}\frac{\partial^{2}\left(w_{b}+w_{z}+{gw}_{z}\right)}{\partial x^{2}}\right)=0$$

(19d) $${-L_{z}\frac{\partial u_{0}}{\partial x}+A}_{s}\left(\frac{\partial^{2}w_{s}}{\partial x^{2}}+\frac{\partial^{2}w_{z}}{\partial x^{2}}\right)+Y_{z}^{b}\frac{\partial^{2}w_{z}}{\partial x^{2}}+Y_{z}^{s}\frac{\partial^{2}w_{z}}{\partial x^{2}}-Z_{33}w_{z}+Y_{z}^{s}\frac{\partial^{2}w_{z}}{\partial x^{2}}+g\left(z\right)q=0$$

### 4.3. Closed-Form Solutions for Simply Supported Beams

For a simply supported type edge, at *x =* 0 and *x = L*, the beam must adhere the following boundary conditions:

(20a) $$w_{b}\left(0\right)=\frac{d^{2}w_{b}\left(0\right)}{dx^{2}}=w_{b}\left(L\right)=\frac{d^{2}w_{b}\left(L\right)}{dx^{2}}=0$$

(20b) $$w_{s}\left(0\right)=\frac{d^{2}w_{s}\left(0\right)}{dx^{2}}=w_{s}\left(L\right)=\frac{d^{2}w_{s}\left(L\right)}{dx^{2}}=0$$

(20c) $$w_{z}\left(0\right)=\frac{d^{2}w_{z}\left(0\right)}{dx^{2}}=w_{z}\left(L\right)=\frac{d^{2}w_{z}\left(L\right)}{{dx}^{2}}=0$$

Navier’s admissible displacement functions in the form of trigonometric series are presented in Equation (21), which are appropriate for such simply supported beams and can be used to solve the governing equations of motion.

(21a) $$u_{0}(x)=\sum_{n=1}^{\infty }U_{n}cos\left(\lambda x\right)$$

(21b) $$w_{b}(x)=\sum_{n=1}^{\infty }W_{bn}sin\left(\lambda x\right)$$

(21c) $$w_{s}(x)=\sum_{n=1}^{\infty }W_{sn}sin\left(\lambda x\right)$$

(21d) $$w_{z}(x)=\sum_{n=1}^{\infty }W_{zn}sin\left(\lambda x\right)$$

where λ *= mπ/L* and ($U_{n},\;W_{bn},\;W_{sn},\;{and\;W}_{zn}$) are the arbitrary parameters to be determined.

**Figure 4:**
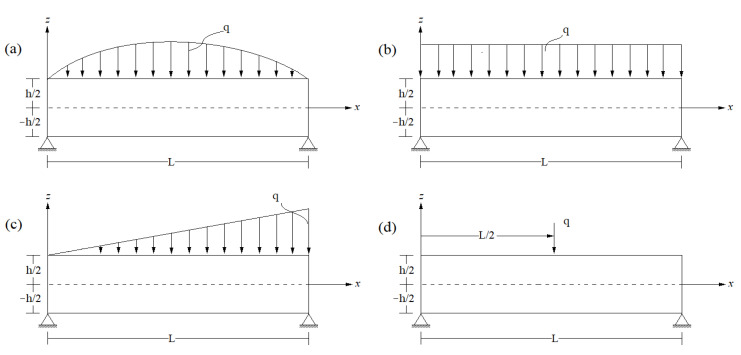
Concrete beams under different load patterns: (**a**) sinusoidal loading; (**b**) uniformly distributed loading; (**c**) linearly distributed loading; (**d**) concentrated loading.

In our analysis, we assume that the transverse bending load “*q*” exhibits various patterns, as illustrated in Figure 4. This bending load can be expanded using the Double Fourier’s single sine series, as follows:

(22) $$q\left(x\right)=\sum_{n=0}^{\infty }Q_{n}sin\left(\lambda x\right)$$

Where the amplitude of each load according to Sayyad et al. [73] is designated in Equation (23):

(23) $$Q_{n}=\left\{\begin{array}{cc} q_{0} & for\;sinusoidal\;load,\;n=1\\ \frac{4q_{0}}{n\pi } & for\;uniformly\;distributed\;load,\;n=1,3,5,\ldots \;\\ -\frac{\sqrt{8}q_{0}}{n\pi }cos\left(n\pi \right) & for\;linearly\;distributed\;load,\;n=1,3,5,\ldots \\ \frac{2q_{0}}{L}sin\left(\frac{n\pi }{2}\right) & for\;concentrated\;load,\;n=1,3,5,\ldots \end{array}\right.$$

Finally, in order to obtain analytical solutions, the results of the substitution can be arranged into the following matrix form:

(24) $$\left[\begin{array}{cccc} S_{11} & S_{12} & S_{13} & S_{14}\\ S_{12} & S_{22} & S_{23} & S_{24}\\ S_{13} & S_{23} & S_{33} & S_{34}\\ S_{14} & S_{24} & S_{34} & S_{44} \end{array}\right]\left\{\begin{array}{c} \begin{array}{c} U_{n}\\ W_{bn}\\ W_{sn} \end{array}\\ W_{zn} \end{array}\right\}=\left\{\begin{array}{c} \begin{array}{c} 0\\ {-Q}_{n}\\ {-Q}_{n} \end{array}\\ {-Q}_{n} \end{array}\right\}$$

where:

(25a) $$S_{11}=-A\lambda^{2};\;S_{12}=B{\lambda^{3}\;;\;S}_{13}={B_{s}\lambda }^{3}\;;\;S_{14}=L\lambda$$

(25b) $$S_{22}={-D\lambda }^{4}-K_{w}-{{K_{s}\lambda }^{2}\;;\;S}_{23}={{-D}_{s}\lambda }^{4}-K_{w}-{K_{s}\lambda }^{2}\;;S_{24}=-Yzb\lambda 2-gzKw+Ks$$

(25c) $$S_{33}={{-H}_{s}\lambda }^{4}-A_{s}\lambda^{2}-K_{w}-{K_{s}\lambda }^{2}\;;S_{34}=-A_{s}\lambda^{2}-Y_{z}^{s}\lambda^{2}-g\left(z\right)\left(K_{w}+K_{s}\right)$$

(25d) $$S_{44}=-A_{s}\lambda^{2}-Z_{33}$$

## 5. Results and Discussion

This Section presents an intriguing exploration of the mechanical bending behavior exhibited by concrete beams infused with an assortment of clay nano-platelets. Through the utilization of the refined quasi-3D beam deformation theory, we embark on the meticulous calculation of transverse displacements ($\bar{w}$), axial displacements ($\bar{U}$), as well as normal and shear stresses ($\sigma_{x}$, $\sigma_{z}$, and $\tau_{xz}$). These comprehensive analyses yield a set of dimensionless equations (Equation (26)), which not only facilitate the interpretation of the obtained results, but also allow their visual representation in numerical and graphical illustrations. By employing these equations, we uncover valuable insights into the static behavior of the beams, providing a deeper understanding of their mechanical response.

(26) $$\begin{array}{c} \bar{w}=\frac{10E_{m}h^{3}}{q_{0}L^{4}}w\left(\frac{L}{2},0\right)\;;\;\bar{U}=\frac{10E_{m}h^{3}}{q_{0}L^{4}}u\left(0,z\right)\;\\ \sigma_{\alpha }\left(z\right)=\frac{h}{q_{0}L}\sigma_{\alpha }^{0}\left(\frac{L}{2},z\right)\;;(\alpha =x,\;z)\\ \tau_{xz}\left(z\right)=\frac{h}{q_{0}L}\tau_{xz}^{0}\left(0,z\right). \end{array}$$

The non-dimensional coefficients of two-parameter (Winkler–Pasternak) elastic foundations are utilized in Equation (27):

(27) $${\bar{k}}_{w}=\frac{L^{4}}{D_{m}}K_{w}\;;\;{\bar{k}}_{s}=\frac{L^{2}}{D_{m}}K_{s}\;;\;D_{m}=\frac{{E_{m}h}^{3}}{12\left(1-{\upsilon_{m}}^{2}\right)}$$

In this analysis, we explore diverse nano-sized clay platelets (which take the form of disk-shaped particles) with different chemical compositions and elastic characteristics to unlock their potential as reinforcements in a concrete matrix. Building upon the insightful work of Wang et al. [74], we are equipped with valuable data on the elastic properties of these remarkable reinforcements, including the bulk modulus (*K_p_*), shear modulus (*G_p_*), and Poisson’s ratio (*υ_p_*), as meticulously compiled in Table 7. To estimate the Young’s modulus of the used reinforcement (*E_p_*), we employ a conversion formula designed for isotropic materials, as expressed in Equation (28):

(28) $$E_{p}=3K_{p}\left(1-2\upsilon_{p}\right)$$

In our study, we aim to create a nano-composite matrix by incorporating nano-platelets into a concrete mixture. The concrete matrix itself possesses specific elastic properties, including an elastic modulus (*E_m_*) of 20 GPa and a Poisson’s ratio (*υ_m_*) of 0.2. By introducing these nano-platelets into the mixture, we seek to enhance the overall mechanical and physical efficiency of the composite material. To evaluate and predict the elastic properties of the resulting nano-composite material, we employ Eshelby’s homogenization approach. This approach allows us to assess the performance of different types of nanometric reinforcement in concrete matrices, enabling the comparative analysis of their effectiveness.

### 5.1. Validation

First, it is highly relevant to consider the accuracy of the current mathematical model based on the refined quasi-3D beam theory assumptions. Since there is a lack of numerical results in the existing literature specifically focusing on the flexural analysis of beams reinforced with clay nano-platelets, we consider the material and geometric properties of Thuc Vo et al. [71] for the flexural analysis of beams made of functionally graded material (FGM). The results in terms of the transverse displacements ($\bar{w}$) as well as normal and shear stresses ($\sigma_{x}$,$\tau_{xz}$) of functionally graded (FG) beams, while varying the power law index (*p*), are implemented and presented in Table 8 for comparison.

The comparison of the results presented in Table 8 was conducted for an FG beam under a uniformly distributed bending load. The findings reveal a remarkable agreement between our current results and those reported by Thuc Vo et al. [71]. Both quasi-3D beam theories accurately predicted the same transverse displacement ($\bar{w}$) and shear stresses ($\sigma_{x}$,$\tau_{xz}$), despite the utilization of different shape functions *f(z)*. This consistency demonstrates the reliability and accuracy of the adopted model in our analysis. On the other hand, when we compared the results obtained from the trigonometric beam theory (TBT) and the first-order beam theory (FBT), it was observed that the vertical displacement ($\bar{w}$) and shear stresses ($\sigma_{x}$,$\tau_{xz}$) slightly exceeded those predicted based on the quasi-3D beam theories. This difference can be attributed to the fact that the quasi-3D theories account for the thickness stretching effect on the beam’s flexural behavior ($\varepsilon_{z}\neq 0$). In contrast, the classical beam theory (CBT) exhibited the lowest accuracy among those of the theories examined.

### 5.2. Simply Supported Beams (without an Elastic Foundation)

**Figure 5:**
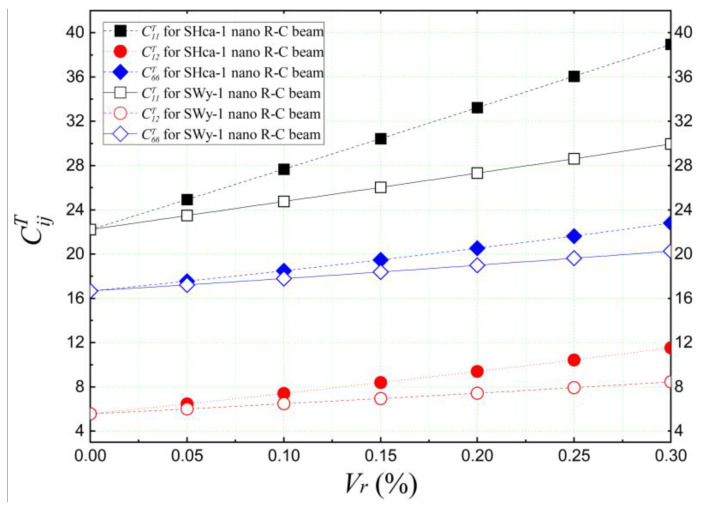
Homogenized elastic stiffnesses $C_{ij}^{T}$ of concrete reinforced with clay nano-platelets.

First, the analytical estimation was conducted using Eshelby’s homogenization approach to determine the reduced elastic constants “$C_{ij}^{T}$” of nano-impregnated concrete and demonstrate the effect of adding clay nanoparticles (NCs) on the elastic properties of the concrete mixtures. Figure 5 illustrates the evolution of elastic stiffnesses “$C_{ij}^{T}$” for nano-incorporated concretes as a function of the volume proportions (*V_r_*) of montmorillonite (SWy-1) and hectorite (SHca-1) nano-platelets. The percentage variations in the added nanometric materials range between 0% and 30% of the total weight of the matrix.

The results shown in Figure 5 indicate that both reinforcements (SWy-1 and SHca-1) have a strengthening effect on the concrete mixture. It is observed that the reduced elastic stiffness ($C_{ij}^{T}$) of the impregnated concrete increases with the concentration of the reinforcement (*V_r_*). Notably, the reduced stiffness “$C_{ij}^{T}$” of the concrete reinforced with hectorite nano-platelets (SHca-1) is significantly higher than that of the montmorillonite (SWy-1) reinforcement, particularly in the case of C_11_, which represents the elastic stiffness in the length-wise direction of the structure. This improvement can be attributed to the superior elastic properties of hectorite nanoparticles.

**Figure 6:**
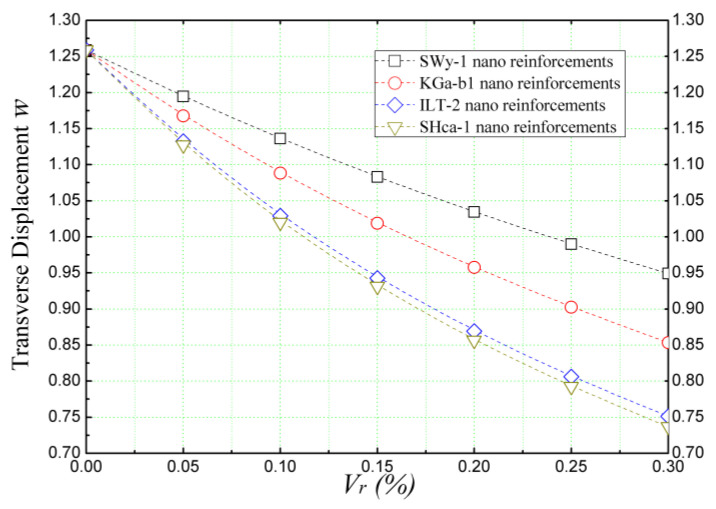
Dimensionless deflection of concrete beams reinforced with different clay nano-platelets (*L/h* = 4, ${\bar{k}}_{w}$ = 0, and ${\bar{k}}_{s}$ = 0).

Additionally, Figure 6 provides an illustration of the impact of volume proportions (*Vr*) of different clay nano-platelets, namely montmorillonite (SWy-1), kaolinite (KGa-1b), illite (ILT-2), and hectorite (Sha-1), on the non-dimensional transverse displacement ($\bar{w}$) of nano-reinforced concrete beams subjected to a sinusoidally distributed load. The findings depicted in Figure 6 clearly demonstrate the strengthening effect of all the clay nano-platelets on the concrete beam. It is evident that the transverse displacement ($\bar{w}$) decreases significantly as the volume proportions of the nanoscale reinforcements in the concrete matrix increase. Specifically, hectorite (SHca-1) nano-platelets exhibit remarkable efficiency as a reinforcement, reducing the non-dimensional vertical displacement ($\bar{w}$) from 1.26 at *V_r_* = 0% (non-reinforced concrete beam) to 0.74 at *V_r_* = 30% (where the reinforcement constitutes 30% of the total weight ‘wt%’). This establishes hectorite as the most effective reinforcement among the various clay nano-platelets considered.

A convergence study is presented in Table 9 in which we investigated the bending behavior of a concrete beam reinforced with hectorite (SHca-1) clay nano-platelets. The presented results listed in Table 3 specifically focuses on examining the effect of the number of terms (*n*) used in Equations (22) and (23) on the accuracy of the calculated transverse displacement of the beam. It is known that for the single sign type of loading, a single term (*n* = 1) is sufficient to generate accurate results. However, for other loading types such as a uniformly distributed load, a linearly distributed load, and a concentrated force, a convergence study is necessary to determine the number of iterations required to achieve accurate results.

As presented in Table 9, the convergence of the linearly distributed load, uniformly distributed load, and concentrated force results was observed in 11 iterations (*n* = 11) using Equations (22) and (23). However, for the subsequent results, a higher number of iterations (*n* = 201) was utilized to obtain more precise and accurate results. This choice was made considering the computational power available, allowing for more thorough calculations and improving the accuracy.

**Figure 7:**
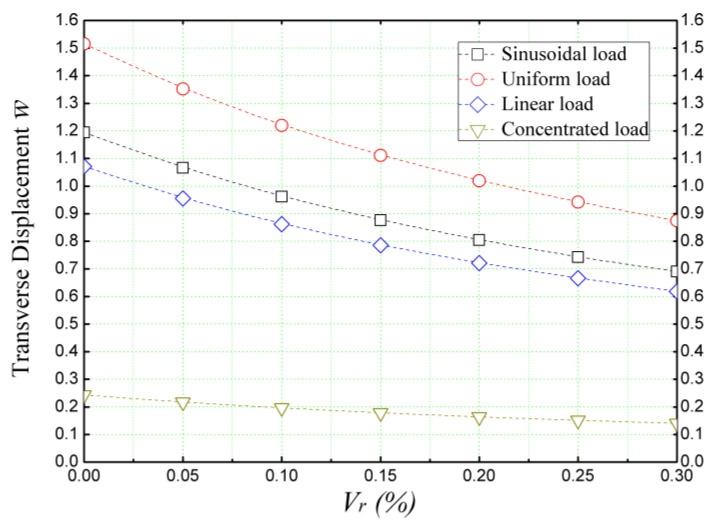
Dimensionless deflection of a concrete beam reinforced with SHca-1 nana-sized platelets and subjected to different bending loads (*L/h* = 10, ${\bar{k}}_{w}$ = 0, and ${\bar{k}}_{s}$ = 0).

Figure 7 depicts the influence of different external bending loads on the transverse displacement ($\bar{w}$) of a concrete beam reinforced with hectorite nanoparticles (SHca-1). The beam was analyzed using the refined quasi-3D theory, while the volume concentrations of the nanoparticles range between 0% (non-reinforced beam) and *V_r_* = 30%. The results obtained reveal that the incorporation of nanoparticles in the concrete matrix leads to a stiffening effect, irrespective of the applied external bending load. Among the various loads examined, the uniform load emerges as the most significant factor influencing the transverse displacement, causing a substantial reduction of ($\bar{w}$).

On the other hand, the concentrated load has the smallest impact on the beam, resulting in a relatively smaller change in transverse displacement. These findings highlight the ability of hectorite nanoparticles to enhance the overall stiffness and performance of the concrete beam under different external bending loads.

**Figure 8:**
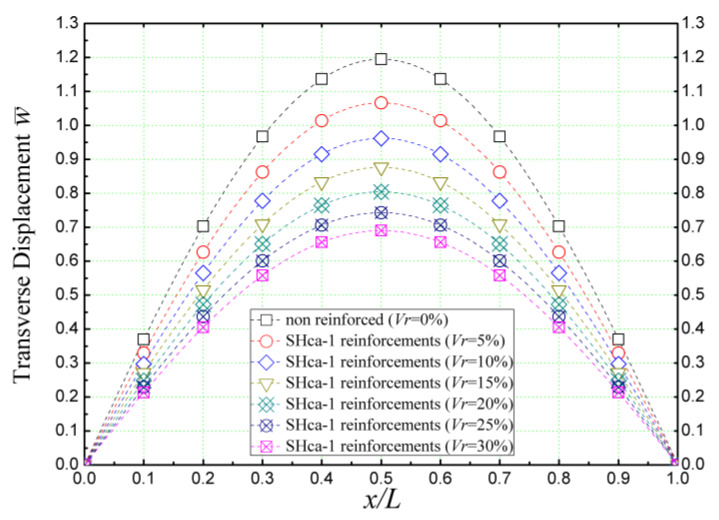
Dimensionless transverse displacement on the entire length of concrete beams reinforced with different proportions of SHca-1 nanoparticles (*L/h* = 10, ${\bar{k}}_{w}$ = 0, and ${\bar{k}}_{s}$ = 0).

Figure 8 presents the impact of incorporating nano-sized hectorite (SHca-1) platelets on the static behavior of concrete beams subjected to single sine loads. The transverse displacement ($\bar{w}$) is evaluated along the entire length of the structure (*x/L*) using the quasi-3D beam deformation theory, which accounts for the thickness stretching effect.

Consistent with previous observations, Figure 8 further demonstrates the reinforcing effect introduced by the inclusion of clay nanoparticles (NC’s) in the concrete structure. Notably, as the volume fraction of impregnated nanoparticles increases, the transverse displacement of the reinforced beam experiences a significant reduction. This reduction in transverse displacement signifies the enhanced stiffness and strength imparted by the presence of hectorite platelets.

It is worth mentioning that the maximum deflection of the beam occurs at the mid-length position, a characteristic that can be attributed to the homogeneity achieved using Eshelby’s homogenization law. This homogeneity plays a pivotal role in distributing the load and deformation evenly along the beam, resulting in a concentrated deflection at the midpoint.

**Figure 9:**
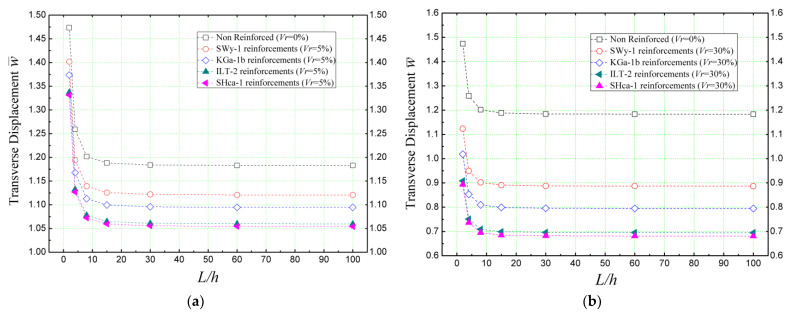
Effect of the geometry parameter (length-to-thickness ratio) on the dimensionless deflection of concrete beams reinforced with clay nano-platelets (${\bar{k}}_{w}$ = 0 and ${\bar{k}}_{s}$ = 0) (**a**): *V_r_* = 5%; (**b**): *V_r_* = 30%.

Figure 9 provides insights into the influence of the geometric ratio (*L/h*), representing the length-to-thickness ratio, on the non-dimensional transverse displacement ($\bar{w}$) of concrete beams reinforced with various clay nano-platelets. The beam is assumed to be simply supported and subjected to sinusoidal loading. The results depicted in Figure 9 clearly demonstrate that hectorite (SHca-1) outperforms the other clay nano-platelets in terms of reducing beam deflection, regardless of the geometric ratios considered. This indicates the superior reinforcing effect of hectorite in minimizing beam deformation.

Furthermore, the comparison between Figure 9a,b reveals that increasing the volume proportions of the reinforcement (*V_r_*) up to 30% leads to a reduction in deflection for the concrete beams. This highlights the importance of reinforcement concentrations in enhancing the structural rigidity and mitigating deflection.

**Figure 10:**
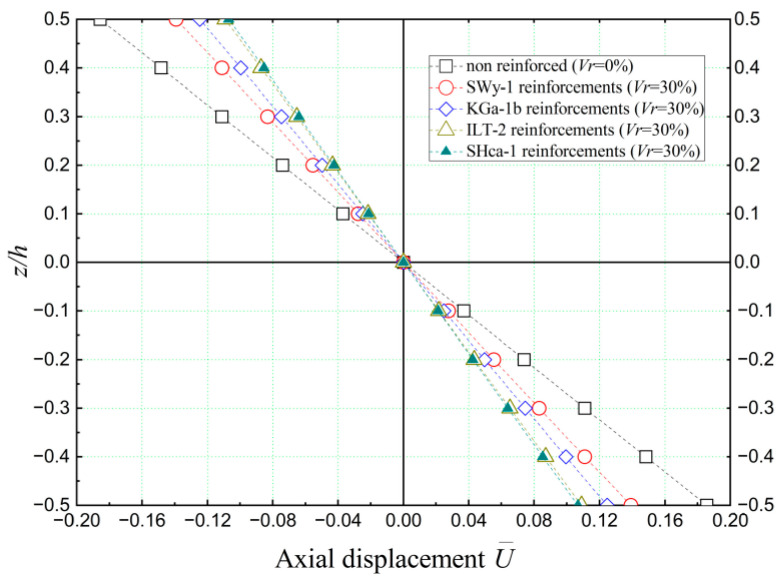
Dimensionless axial displacement ($\bar{U}$) of non-reinforced and nano-clay-reinforced concrete beams (*L/h* = 10, *V_r_* = 30%, ${\bar{k}}_{w}$ = 0, $and\;{\bar{k}}_{s}$ = 0).

Figure 10 presents the axial displacements ($\bar{U}$) of concrete beams reinforced with nano-sized clay platelets when subjected to sinusoidal loading. Various types of clay nano-platelets were considered in this analysis.

A notable observation from Figure 10 is that the axial displacements exhibit symmetry and have a value of 0 at the median plane (*z* = 0) of the beams. This symmetry is attributed to the achieved homogeneity between the reinforcements and the matrix, accomplished through the utilization of Eshelby’s homogenization approach, as well as the assumptions made regarding the distribution of clay nano-platelets within the concrete matrix.

Furthermore, it is worth noting that the maximum axial displacement ($\bar{U}$) is observed in the case of the non-reinforced concrete beam (*V_r_* = 0%). This signifies the relatively higher deformation experienced by the non-reinforced beam. Conversely, the minimum axial displacement value is observed when the concrete beam is reinforced with hectorite clay nano-platelets. This suggests that the presence of hectorite reinforcement leads to the significant reduction of axial displacement, indicating its effectiveness in enhancing the stiffness and structural integrity of the concrete beam.

**Figure 11:**
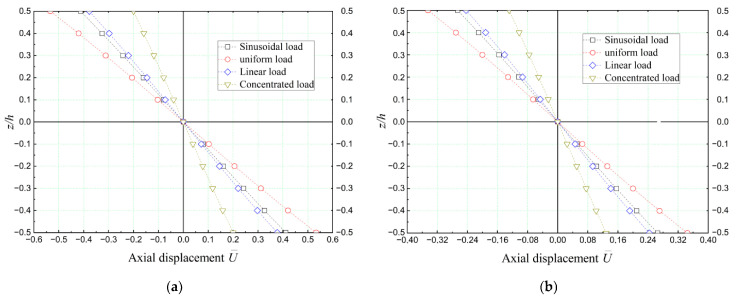
Dimensionless axial displacement ($\bar{U}$) of nano-clay (SHca-1)-reinforced concrete beams subjected to various loads (*L/h* = 4, ${\bar{k}}_{w}$ = 0, and ${\bar{k}}_{s}$ = 0) (**a**): *V_r_* = 5%; (**b**): *V_r_* = 30%.

Figure 11 showcases the impact of various bending load patterns on the non-dimensional axial displacement ($\bar{U}$) of a simply supported concrete beam reinforced with hectorite (SHca-1) nano-sized platelets. The analysis utilizes the quasi-3D beam deformation theory.

Upon comparing Figure 11a with Figure 11b, it becomes evident that the volume concentration of nano-clay platelets plays a crucial role in reducing the non-dimensional axial displacement ($\bar{U}$) of the nano-reinforced concrete beams. This reduction was observed regardless of the specific external mechanical load applied to the beams.

Table 10 provides insights into the influence of various types and proportions of clay nano-reinforcements (NC’s) on the non-dimensional normal and shear stresses ($\sigma_{x}$, $\sigma_{z}$, and $\tau_{xz}$) of simply supported concrete beams under sinusoidally distributed loads. In the analysis, we employ the quasi-3D beam theory, which accounts for the stretching effect ($\varepsilon_{z}\neq 0$) that occurs across the beam’s thickness. A notable observation from Table 10 is that the nano-reinforcements have a tendency to increase the normal stress ($\sigma_{x}$) along the length of the beam and the normal stress ($\sigma_{z}$) across its thickness. This increase in normal stresses can be attributed to the improved elastic properties of the concrete resulting due to the addition of nano-reinforcements. The presence of clay nano-platelets enhances the resistance of the beam in both its primary direction (length) and thickness, leading to a decreased transverse displacement ($\bar{w}$) and increased normal stresses ($\sigma_{x}$ and $\sigma_{z}$). Conversely, the shear stress ($\tau_{xz}$) experiences a slight decrease due to the inclusion of nano-reinforcements. This suggests that the nano-clay platelets contribute to a redistribution of stresses within the beam, resulting in reduced shear stress levels across its thickness.

The findings in Table 10 shed light on the impact of different clay nano-reinforcements and their proportions on the non-dimensional normal and shear stresses of simply supported concrete beams. This understanding is crucial for optimizing the design and performance of nano-reinforced concrete structures.

### 5.3. The Effect of Winkler–Pasternak’s Elastic Foundation

**Figure 12:**
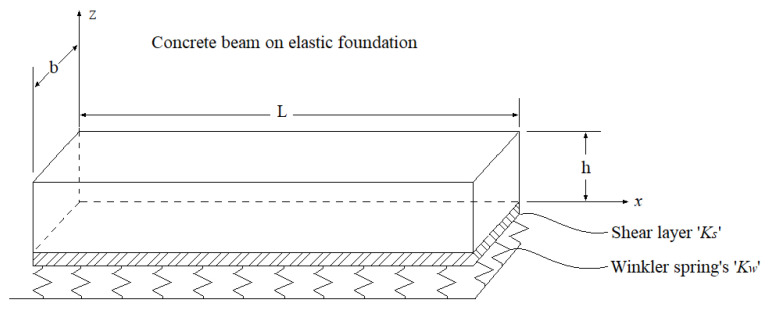
A concrete beam resting on Winkler–Pasternak’s elastic foundation.

To enhance the scope of our study, we expanded upon our analysis to consider the scenario where the nano-clay (NC)-reinforced concrete beam is supported by an elastic foundation. Figure 12 illustrates the configuration of a nano-clay-reinforced concrete beam resting on a Winkler–Pasternak elastic foundation. This specific type of elastic foundation comprises a shear layer with a stiffness constant of *K_s_*, which is connected to springs with a stiffness constant of *K_w_*.

In the subsequent analysis, we investigate the impact of various parameters associated with the Winkler–Pasternak elastic foundation on the static response, including the transverse displacements, normal stresses, and shear stresses of concrete beams reinforced with different types of clay nano-platelets.

**Figure 13:**
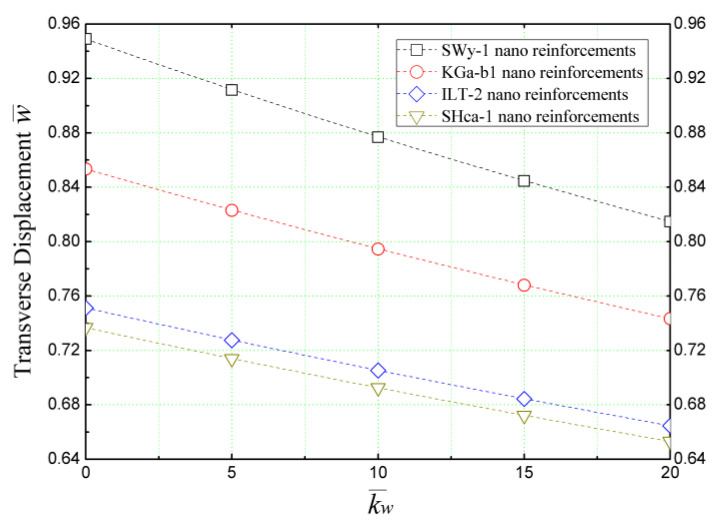
The effect of Winkler springs constant (${\bar{k}}_{w}$) on the dimensionless transverse displacement of concrete beams reinforced with several types of clay nano-platelets (*L/h* = 4 and ${\bar{k}}_{s}$ = 0).

Figure 13 depicts the influence of Winkler’s parameter, represented by the springs constant “${\bar{k}}_{w}$“, on the transverse displacement ($\bar{w}$) of concrete beams reinforced with various clay nanoparticles. The results presented in Figure 13 demonstrate that an increase in the Winkler parameter “${\bar{k}}_{w}$“ enhances the resilience of the reinforced beam under external sinusoidal loading, leading to the reduced bending of the concrete structure. Consistent with previous findings, it is noteworthy that hectorite nano-platelets exhibit the most significant effect compared to those of other types of reinforcements.

**Figure 14:**
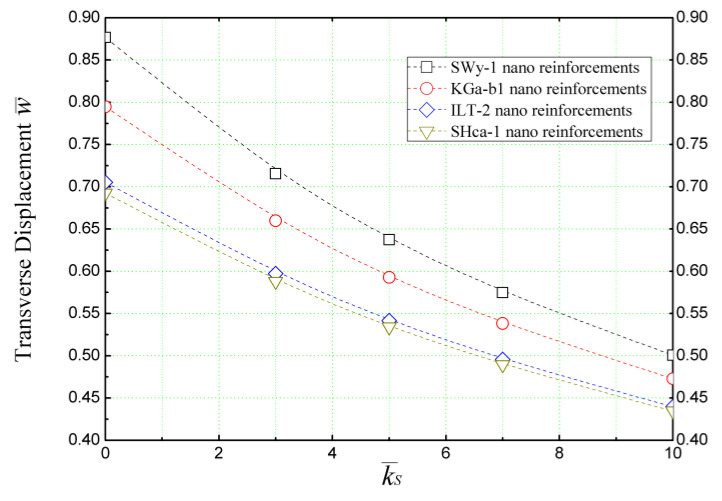
The effect of the shear layer parameter (${\bar{k}}_{s}$) of Winkler/Pasternak’s elastic foundation on nano-clay-reinforced concrete beams (*L/h* = 4 and ${\bar{k}}_{w}$ = 10).

Taking into account both the shear layer constant and the spring constant, we examine the impact of the Winkler–Pasternak foundation on the transverse displacement of a nano-clay-reinforced concrete beam in Figure 14. The beam is subjected to a sinusoidal bending load, and the calculations are carried out using the quasi-3D beam theory, which accounts for the stretching effect in the thickness direction.

Figure 14 illustrates that the shear constant, represented by ${\bar{k}}_{s}$, significantly influences the non-dimensional transverse displacement ($\bar{w}$) of the beam by reducing its deflection. The magnitude of this effect varies depending on the type of nano-reinforcement employed in the concrete matrix.

Lastly, Table 11 presents the influence of the elastic constants ${\bar{k}}_{w}$ and ${\bar{k}}_{s}$ of the Winkler–Pasternak foundation on the non-dimensional normal stresses ($\sigma_{x}$ and $\sigma_{z}$), and shear stress ($\tau_{xz}$), considering various types of clay nano-reinforcements in the concrete beam. Upon examining the results listed in Table 11, it is worth noting that all the clay nano-reinforcements analyzed in our study exhibit a similar effect on the non-dimensional normal stresses ($\sigma_{x}$ and $\sigma_{z}$) and shear stress ($\tau_{xz}$). They contribute to the reduction of these stresses in the concrete beam. This reduction can be attributed to the distribution of stresses provided by the elastic foundation supporting the beam. Additionally, the elastic foundation, represented by the elastic constants ${\bar{k}}_{w}$ and ${\bar{k}}_{s}$, also contributes to decreasing the bending stresses in the concrete beam.

## 6. Conclusions

Clay minerals are widely abundant in the Earth’s crust and offer versatile applications due to their prevalence and cost-effective extraction. In this study, we delved into the potential of utilizing nanoscale clay additives to improve the elastic and physical properties of concrete beams. To investigate this, we applied the quasi-3D beam theory, which is used to consider the effects of thickness stretching, to analyze the static behavior of concrete beams reinforced with nano-scale clay (NC) additives. By examining the performance of these nano-clay-reinforced beams, we aim to uncover the benefits and implications of incorporating clay additives to enhance the properties of concrete structures.

Our investigation has yielded valuable insights and led us to the following noteworthy conclusions:

1. The addition of nano-sized clay platelets to concrete mixtures results in improved elastic characteristics, with the extent of the improvement being dependent on the volume concentration of these additives.
2. Concrete structures reinforced with nano-clays exhibit exceptional optimization under external mechanical loads, enhancing their overall performance.
3. When they are subjected to various external bending loads, concrete beams reinforced with clay nano-platelets demonstrate a significantly reduced transverse displacement (deflection), highlighting the efficacy of nano-reinforcements in minimizing structural deformations.
4. Among the various types of clay nano-platelets considered, hectorite (SHca-1) is particularly effective in reducing transverse displacements, showcasing a reduction of approximately 42%. This performance surpasses those of illite (ILT-2), with a reduction of 39%, kaolinite (KGa-1b), with a reduction of 32%, and montmorillonite (SWy-1), with a reduction of 24%, in transverse displacement. These findings highlight the superior performance of hectorite nano-platelets in enhancing the structural stability and minimizing deformations in concrete beams.
5. Incorporating an elastic foundation further contributes to stress reduction and deflection mitigation in nano-reinforced concrete beams, enhancing their overall structural integrity.

However, it is important to recognize the limitations of our research, which exclusively centered on nano-clay platelets. In order to expand our understanding and encompass a broader range of structural elements, future investigations should delve into the effects of other nano-reinforcements on the properties of concrete beams, plates, walls, and other structural components. These endeavors should encompass analytical analyses to thoroughly evaluate the influence of these alternative nano-reinforcements. In doing so, we can gain a more comprehensive understanding of their potential and pave the way for further advancements in the field of reinforced concrete structures.

## Figures and Tables

**Table 1:** Chemical composition of nano-clays [47,48].

Components	SiO_2_	Al_2_O_3_	CaO	TiO_2_	Fe_2_O_3_	K_2_O	LOI
**Percentages**	45.5~89.6	18.9~42.3	0.17~3.59	0.82~1.82	0.82~1.82	0.1~10.9	0.03~4.55

**Table 2:** Physical characteristics of nano-clay [50,51,52].

Specimen	Particle Size (nm)			Mean Size (nm)	Surface Area (m²/g)	Percentage of Nanoparticles (<500 nm)
	D10, Effective Size	D30	D60			
**Clay**	1612	5585	13,325	1202	18.231	-
**Nano-clay**	176	395	767	1114	48~170	40%

**Table 3:** QXDA results for cement paste (C) and cement nanocomposite containing 1 wt% NC (NCC1) [47,48].

Phase		Portlandite	Ettringite	Tri-Calcium Silicate	Di-Calcium Silicate	Gypsum	Calcite	Quartz	Amorphous Content
**weight %**	C	16.8	2	1.3	4.4	0.7	3.7	0.9	70.1
	NCC1	13.8	1.6	1.5	6.1	0.4	3	0.5	73

**Table 4:** The effect of clay nanoparticles on compressive strength of normal concrete at 28 days.

Cementitious Materials	w/b Ratio	Concentration of NC (%)	Compressive Strength Increment	Best Content %	Ref.
**Normal concrete**	0.48	2–5	5.27%→15.45%	5	[57]
	0.5	10	26.32%	-	[58]
	0.53	3–10	42.2%→63.1%	10	[59]

**Table 5:** Splitting tensile strength of ordinary concrete infused with clay nanoparticles at 28 days.

Cementitious Materials	w/b Ratio	Concentration of NC (%)	Splitting Tensile Strength Increment %	Best Content %	Ref.
**Normal concrete**	0.5	10	25.87%	-	[58]
	0.53	3–10	0%→46.6%	10	[59]

**Table 6:** Coefficient of capillary absorption of cementitious composites mixed with NC.

Cementitious Materials	Cont. of NC (%)	Capillary Absorption Coefficient		Reduced by (%)	Ref
		Control Sample	Sample with NC		
**Normal concrete**	3–10	-	-	16.6%→25.6%	[59]
**Concrete mortar**	2–14	0.33 (Kg/m^2^)/min^−1/2^	0.12–0.22 (Kg/m^2^)/min^−1/2^	33%→63.6%	[61]
	5–10	-		38%→51.3%	[63]
	2–10	0.056 (Kg/m^2^)/min^−1/2^	0.014–0.04 (Kg/m^2^)/min^−1/2^	28.5%→25.6%	[64]

**Table 7:** Elastic characteristics of used clay nano-platelets. (Wang [74]).

Name of Reinforcement	Montmorillonite	Kaolinite, Well Crystallized	Illite	Hectorite, a Mg-Rich Montmorillonite
**Code**	SWy-1	KGa-1b	ILT-2	SHca-1
$\mathrm{Density}\;‘\rho_{r}’$ (Kg/m^3^)	2600	2444	2706	2667
**Bulk modulus** ‘Kp’ (GPa)	29.7	47.9	60.1	63.4
**Shear modulus** ‘Gp’ (GPa)	16.4	19.7	25.3	26.2
**Poisson’s ratio** ‘υp’	0.267	0.319	0.315	0.318
**Elastic modulus** ‘Ep’ (GPa)	41.5	52	66.7	69.2

**Table 8:** Validation of the current theory with other theories in the literature (*L*/*h* = 5).

Method	*p* = 0			*p* = 1			*p* = 2		
	$\bar{w}$	$\sigma_{x}$	$\tau_{xz}$	$\bar{w}$	$\sigma_{x}$	$\tau_{xz}$	$\bar{w}$	$\sigma_{x}$	$\tau_{xz}$
**Quasi-3d Present**	3.1397	3.8006	0.7233	6.1338	5.8812	0.7233	7.8606	6.8819	0.6622
**Quasi-3d** [71]	3.1397	3.8005	0.7233	6.1338	5.8812	0.7233	7.8606	6.8818	0.6622
**TBT** [75]	3.1657	3.8020	0.7500	6.2599	5.8837	0.7500	8.0602	6.8812	0.6787
**FBT** [75]	3.1657	3.7500	0.5976	6.2599	5.7959	0.5976	8.0303	6.7676	0.5085
**CBT** [75]	2.8783	-	-	5.7746	-	-	7.4003	-	-

**Table 9:** Convergence for the non-dimensional deflection of concrete beam (*L*/*h* = 10) infused with hectorite clay nano-platelets (*Vr* = 20%) under different bending loads.

Loading Type	$\mathrm{Non}-\mathrm{Dimensional}\;\mathrm{Transverse}\;\mathrm{Displacement}\;\bar{w}$									
	*n* = 1	*n* = 3	*n* = 5	*n* = 7	*n*= 9	*n* = 11	*n* = 13	*n* = 15	*n* = 17	*n* = 19
**Uniformly distributed load**	0.8784	0.8744	0.8748	0.8747	0.8748	0.8747	0.8747	0.8747	0.8747	0.8747
**Linearly distributed load**	0.6211	0.6183	0.6186	0.6185	0.6185	0.6185	0.6185	0.6185	0.6185	0.6185
**Concentrated load**	0.1380	0.1399	0.1401	0.1402	0.1403	0.1403	0.1403	0.1403	0.1403	0.1403

**Table 10:** Non-dimensional normal stresses ($\sigma_{x}\;\mathrm{and}\;\sigma_{z}$ ), and shear stress ($\tau_{xz}$ ) of a simply supported concrete beam reinforced with different proportions of clay nano-platelets, (*L/h* = 4, ${\bar{k}}_{w}$ = 0, and ${\bar{k}}_{s}$ = 0).

***V_r_***	Nano Montmorillonite			Nano Kaolinite			Nano Illite			Nano Hectorite		
	$\sigma_{x}$	$\sigma_{z}$	$\tau_{xz}$	$\sigma_{x}$	$\sigma_{z}$	$\tau_{xz}$	$\sigma_{x}$	$\sigma_{z}$	$\tau_{xz}$	$\sigma_{x}$	$\sigma_{z}$	$\tau_{xz}$
0%	0.6150	0.3924	1.1927	0.6150	0.3924	1.1927	0.6150	0.3924	1.1927	0.6150	0.3924	1.1927
5%	0.6152	0.3927	1.1926	0.6153	0.3929	1.1926	0.6155	0.3929	1.1926	0.6155	0.3929	1.1926
10%	0.6153	0.3930	1.1926	0.6155	0.3935	1.1926	0.6159	0.3934	1.1925	0.6159	0.3934	1.1925
15%	0.6154	0.3933	1.1926	0.6157	0.3941	1.1926	0.6162	0.3939	1.1925	0.6163	0.3939	1.1925
20%	0.6155	0.3936	1.1926	0.6159	0.3946	1.1925	0.6165	0.3943	1.1925	0.6166	0.3944	1.1924
25%	0.6156	0.3940	1.1926	0.6161	0.3952	1.1925	0.6168	0.3948	1.1924	0.6169	0.3949	1.1924
30%	0.6157	0.3943	1.1926	0.6162	0.3959	1.1925	0.6169	0.3953	1.1924	0.6171	0.3954	1.1924

**Table 11:** The effect of Winkler/Pasternak’s elastic foundation on the non-dimensional normal stresses ($\sigma_{x}$ and $\sigma_{z}$), and shear stress ($\tau_{xz}$) of nano-clay-reinforced concrete beam (*L/h* = 4 and *V_r_* = 30%).

(${\bar{k}}_{w}$, ${\bar{k}}_{s}$)	**nano Montmorillonite**			**nano Kaolinite**		
	$\sigma_{x}$	$\sigma_{z}$	$\tau_{xz}$	$\sigma_{x}$	$\sigma_{z}$	$\tau_{xz}$
(10, 0)	0.5695	0.3648	1.1018	0.5744	0.3690	1.1103
(10, 5)	0.4156	0.2662	0.8010	0.4303	0.2764	0.8283
(10, 10)	0.3272	0.2096	0.6292	0.3439	0.2210	0.6606
(20, 10)	0.3137	0.2009	0.6030	0.3305	0.2123	0.6345
(${\bar{k}}_{w}$, ${\bar{k}}_{s}$)	**nano Illite**			**nano Hectorite**		
	$\sigma_{x}$	$\sigma_{z}$	$\tau_{xz}$	$\sigma_{x}$	$\sigma_{z}$	$\tau_{xz}$
(10, 0)	0.5797	0.3715	1.1194	0.5805	0.3720	1.1207
(10, 5)	0.4468	0.2863	0.8597	0.4493	0.2879	0.8643
(10, 10)	0.3635	0.2329	0.6978	0.3664	0.2348	0.7034
(20, 10)	0.3502	0.2244	0.6721	0.3532	0.2263	0.6778

## Data Availability

The data presented in this study are available on request from the corresponding author.
